# Immobilization of pamidronic acids on the nanotube surface of titanium discs and their interaction with bone cells

**DOI:** 10.1186/1556-276X-8-124

**Published:** 2013-03-12

**Authors:** Tae-Hyung Koo, Jyoti S Borah, Zhi-Cai Xing, Sung-Mo Moon, Yongsoo Jeong, Inn-Kyu Kang

**Affiliations:** 1 Department of Polymer Science and Engineering, Kyungpook National University, Daegu, 702-701, South Korea; 2 Department of Surface Technology, Korea Institute of Material Science, Changwon-si, 642-831, South Korea

**Keywords:** Pamidronic acid, TiO_2_ nanotubes, Immobilization, Surface modification, Bone cell

## Abstract

Self-assembled layers of vertically aligned titanium nanotubes were fabricated on a Ti disc by anodization. Pamidronic acids (PDAs) were then immobilized on the nanotube surface to improve osseointegration. Wide-angle X-ray diffraction, X-ray photoelectron microscopy, and scanning electron microscopy were employed to characterize the structure and morphology of the PDA-immobilized TiO_2_ nanotubes. The *in vitro* behavior of osteoblast and osteoclast cells cultured on an unmodified and surface-modified Ti disc was examined in terms of cell adhesion, proliferation, and differentiation. Osteoblast adhesion, proliferation, and differentiation were improved substantially by the topography of the TiO_2_ nanotubes, producing an interlocked cell structure. PDA immobilized on the TiO_2_ nanotube surface suppressed the viability of the osteoclasts and reduced their bone resorption activity.

## Background

The clinical success of orthopedic and dental implants depends on the interaction between the implanted surface and bone tissues and, consequently, their osseointegration [1]. Titanium implants are used widely in orthopedic surgery and dentistry for their favorable biocompatibility and corrosion resistance [2,3]. Surface modification of the implanted material is a critical factor for tissue acceptance and cell survival. Among three different crystalline phases of titania (anatase, rutile, and amorphous titania), anatase phase is more favorable for cell adhesion and proliferation due to lower surface contact angles and/or wettability [4]. Several surface modification techniques, i.e., sol–gel techniques, chemical (alkali/acid) treatment, anodization, plasma spray, hydroxyapatite-coated surface, and self-assembled monolayers, have been developed and are currently used with the aim of enhancing the bioactivity of pure Ti surface [5-12].

Over the last decade, bisphosphonates (BPs) have attracted increasing attention as a surface modifier for orthopedic and dental implants. Bisphosphonates are stable pyrophosphates that prevent the loss of bone mass and are used widely to treat a range of diseases with excess bone resorption, such as bone metastasis, hypercalcemia of a malignancy, and Paget’s disease [13-16]. In orthopedic implants, the use of BP is expected to promote osteogenesis at the bone tissue/implant interface by inhibiting the activity of osteoclasts. BPs were reported to inhibit the differentiation of the osteoclast precursor and the resorptive activity of mature osteoclasts [17,18]. Furthermore, BPs alter the morphology of osteoclasts, such as a lack of ruffled border and disruption of the actin ring, both *in vitro* and *in vivo*[19,20]. García-Moreno et al. reported that BPs enhance the proliferation, differentiation, and bone-forming activity of osteoblasts directly [21]. Recently, pamidronic acid, a nitrogen-containing bisphosphonate, was reported to conjugate the titanium surface and stimulate new bone formations around the implant both *in vitro* and *in vivo*, which contribute to the success of the implant technology [22,23].

Besides chemical surface modifications, nanometric-scale surface topography and roughness of the biomaterial is also recognized as a critical factor for tissue acceptance and cell survival. Nanoscale topography affects cell adhesion and osteoblast differentiation [24-26]. It was reported that the fabrication of TiO_2_ nanotubes on titanium implants increased new bone formation significantly [27]. To study the effect of the nanopore size on bone cell differentiation and proliferation, Park et al. used vertically aligned TiO_2_ nanotubes with six different diameters between 15 and 100 nm. They reported 15 nm to be the optimal length scale of the surface topography for cell adhesion and differentiation [28]. TiO_2_ nanotubes can modulate the bone formation events at the bone-implant interface to reach a favorable molecular response and osseointegration [29]. Immobilization of bone morphogenetic protein 2 (BMP-2) on TiO_2_ nanotubes stimulates both chondrogenic and osteogenic differentiation of mesenchymal stem cells (MSCs). Surface-functionalized TiO_2_ nanotubes with BMP-2 synergistically promoted the differentiation of MSCs [30,31]. Furthermore, TiO_2_ nanotubes can control the cell fate and interfacial osteogenesis by altering their nanoscale dimensions, which have no dependency or side effects [32].

In this study, dual-surface modifications, i.e., nanometric-scale surface topography and chemical modification were examined to improve the osteogenesis of titanium implants. First, TiO_2_ nanotubes were fabricated on a Ti disc and pamidronic acid (PDA) was then immobilized on the nanotube surface. The behavior of osteoblasts and osteoclasts on the dual-surface modified and unmodified Ti disc surface were compared in terms of cell adhesion, proliferation, and differentiation to examine the potential for use in bone regeneration and tissue engineering. The motivation for the immobilization of PDA on nanotube surface was that PDA, a nitrogen-containing bisphosphonate, suppresses the osteoclast activity and improves the osseointegration of TiO_2_ nanotubes.

## Methods

### Nanotube formation

TiO_2_ nanotubes were prepared on a Ti disc surface by an anodizing method in a two-electrode (distance between the two electrodes is 7 cm) electrochemical cell with platinum foil as the counter electrode at a constant anodic potential of 25 V and current density of 20 V, in a 1 M H_3_PO_4_ (Merck, Whitehouse Station, NJ, USA) and 0.3 wt.% HF (Merck) aqueous solution with 100-rpm magnetic agitation at 20°C. The Ti disc specimen was commercially pure titanium grade IV. The specimen was cleaned ultrasonically in ethanol for 10 min and chemically polished in a 10 vol.% HF and 60 vol.% H_2_O_2_ solution for 3 min. All electrolytes were prepared from reagent-grade chemicals and deionized water. Heat treatment of TiO_2_ nanotubes was carried out for 3 h at 350°C in air. The morphology of the TiO_2_ nanotubes was observed by field emission scanning electron microscopy (FE-SEM; JSM 6700F, Jeol Co., Akishima-shi, Japan), and their crystal structure was analyzed by wide-angle X-ray diffraction (WAXD, PANalytical’s X’PertPro, Almelo, The Netherlands).

### Immobilization of PDA on a nt-TiO_2_ disc

**Figure 1:**
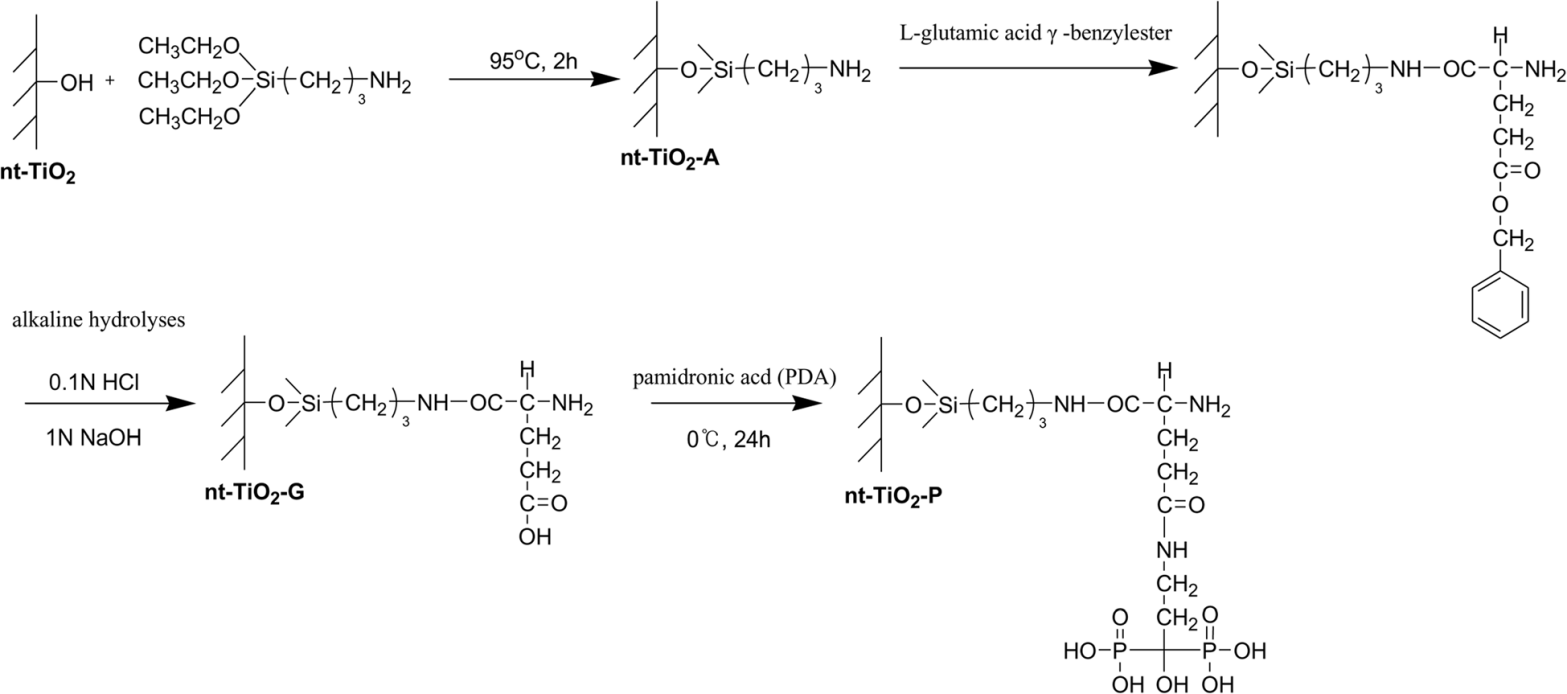
**Schematic diagram showing the PDA-immobilized TiO**_**2 **_**nanotubes.**

The immobilization of PDA on the TiO_2_ nanotube (nt-TiO_2_) disc was carried out in three steps. First, the carboxyl group (−COOH) was introduced to the nt-TiO_2_ disc surface by a reaction of aminopropyl triethoxysilane (APTES; Sigma-Aldrich, St. Louis, MO, USA) with l-glutamic acid γ-benzyl ester (Sigma-Aldrich) followed by alkaline hydrolysis. Subsequently, PDA was immobilized on the carboxyl groups of the nt-TiO_2_ disc surface using water-soluble carbodiimide (WSC). Briefly, a nt-TiO_2_ disc (1 × 1 cm^2^) was immersed in an APTES-water solution (1:9) and sonicated for 30 min. The disc was then heated to 95°C for 2 h with gentle stirring. The silanized nt-TiO_2_ disc was washed with water in an ultrasonic cleaner and dried under reduced pressure and room temperature to produce a primary amine-coupled TiO_2_ nanotube disc (nt-TiO_2_-A). The nt-TiO_2_-A was then immersed in a beaker containing aqueous solution of l-glutamic acid γ-benzyl ester (23.93 mg in 100 ml water) and WSC solution (1-ethyl-3-(3-dimethylaminopropyl)carbodiimide hydrochloride (0.5 g, 0.25 wt.%; Sigma-Aldrich) and *N*-hydroxysuccinimide (0.5 g, 0.25 wt.%; Sigma-Aldrich) dissolved in 20 ml water) and stirred gently for 5 h at 4°C followed by alkaline hydrolysis to obtain the carboxyl functional TiO_2_ nanotube disc (nt-TiO_2_-G). The nt-TiO_2_-G was immersed in a solution of pamidronic acid disodium salt hydrate (10^−4^ M, 100 ml; Sigma-Aldrich) and WSC and stirred gently for 12 h at 4°C to obtain a PDA-immobilized nt-TiO_2_ disc (nt-TiO_2_-P; Figure 1). The nt-TiO_2_-P was then washed in distilled water and dried. The chemical composition of the nt-TiO_2_-P surface was analyzed by electron spectroscopy for chemical analysis (ESCA, ESCA LAB VIG Microtech, East Grinstead, UK) using Mg Kα radiation at 1,253.6 eV and a 150-W power mode at the anode.

### Osteoblastic cell culture

To examine the interaction of the surface-modified and unmodified TiO_2_ discs (Ti, nt-TiO_2_, and nt-TiO_2_-P) with osteoblasts (MC3T3-E1), the circular TiO_2_ discs were fitted to a 24-well culture dish and immersed in a Dulbecco’s modified Eagle’s medium (DMEM) containing 10% fetal bovine serum (FBS; Gibco, Invitrogen, Carlsbad, CA, USA). Subsequently, 1 mL of the MC3T3-E1 cell solution (3 × 10^4^ cells/mL) was added to the TiO_2_ disc surfaces and incubated in a humidified atmosphere containing 5% CO_2_ at 37°C for 4 h, 2 days, 3 days, and 4 days. After incubation, the supernatant was removed and the TiO_2_ discs were washed twice with phosphate-buffered silane (PBS; Gibco) and fixed in a 4% formaldehyde aqueous solution for 15 min. The samples were then dehydrated, dried in a critical-point drier, and sputter-coated with gold. The surface morphology of the TiO_2_ disc was observed by FE-SEM.

To examine the cytotoxic effects of PDA, after 2 days of culture, the osteoblast cells were suspended in PBS with a cell density of 1 × 10^5^ to 1 × 10^6^ cells/mL. Subsequently, 200 μL of a cell suspension was mixed with a 100-μL assay solution (10 μL calcein-AM solution (1 mM in DMSO) and 5 μL propidium iodide (1.5 mM in H_2_O) was mixed with 5 mL PBS) and incubated for 15 min at 37°C. The cells were then examined by fluorescence microscopy (Axioplan 2, Carl Zeiss, Oberkochen, Germany) with 490-nm excitation for the simultaneous monitoring of viable and dead cells.

The proliferation of osteoblasts on the Ti, nt-TiO_2_ and nt-TiO_2_-P discs was determined by a 3-(4,5-dimethylazol-2-yl)-2,5-diphenyl-2H-tetrazolium bromide (MTT) assay. Briefly, MC3T3-E1 osteoblasts were seeded at a concentration of 3 × 10^4^ cells/mL on the Ti, nt-TiO_2_, and nt-TiO_2_-P disc surfaces, which fitted in a 24-well plate, and cell proliferation was monitored after 2 and 3 days of incubation. A MTT solution (50 μL, 5 mg/mL in PBS) was added to each well and incubated in a humidified atmosphere containing 5% CO_2_ at 37°C for 4 h. After removing the medium, the converted dye was dissolved in acidic isopropanol (0.04 N HCl-isopropanol) and kept for 30 min in the dark at room temperature. From each sample, the medium (100 μL) was taken, transferred to a 96-well plate, and subjected to ultraviolet measurements for the converted dye at a wavelength of 570 nm on a kinetic microplate reader (ELx800, Bio-Tek® Instruments, Inc., Highland Park, VT, USA).

The calcium deposition of MC3T3-E1 cells cultured was studied by Alizarin Red S staining. The cells were cultured for 15 days on Ti, nt-TiO_2_, and nt-TiO_2_-P discs under the same condition as described earlier. After incubation, the cells were washed with PBS, fixed in 10% formaldehyde for 30 min, and then triple washed with distilled water for 10 min. The samples were then treated with Alizarin Red S stain solution (1 mL) and incubated for 20 min. After washing the sample with distilled water four times, the digital images of the stained cultures were obtained (Nikon E 4500, Shinjuku, Japan).

### Differentiation of macrophage

For osteoclastic differentiation, hematopoietic stem cells (HSC, name of cell line) at a cell density of 3 × 10^4^ cells/mL were cultivated on Ti, nt-TiO_2_, and nt-TiO_2_-P discs in DMEM containing 10% FBS, 50 ng/mL mouse recombinant receptor activator of nuclear factor kappa-B ligand (RANKL), and 50 ng/mL macrophage colony-stimulating factors from mouse (m-CSF). The culture medium was changed every 2 days.

### Tartrate-resistant acid phosphatase staining and solution assays

To analyze osteoclastic differentiation, the cells after 4 days of culture in the differentiation medium were washed once with PBS and fixed with 10% formalin (50 μL, neutral buffer) at room temperature for 5 min. After fixation, cells were washed with distilled water and incubated with a substrate solution (3 mg of chromogenic substrate with 5 mL tartrate-containing buffer (pH 5.0)) for 30 min at 37°C. The cell images were obtained by fluorescence microscopy.

For immunocytochemistry, the HSCs were cultivated in a differentiation medium and fixed and immunostained after 4 days with 4^′^,6-diamidino-2-phenylindole (DAPI) and (tetra-methyl rhodamine isothiocyanate)-phalloidin (TRICK), as described previously [33]. Multinucleated cells containing more than three nuclei were considered differentiated osteoclast-like cells. The cell images were obtained by fluorescence microscopy. To confirm the viability of the differentiated macrophages on nt-TiO_2_ and nt-TiO_2_-P, the cells after 4 days of culture were stained with calcein-AM and propidium iodide, as described in the section for the osteoblastic cell culture, and examined by fluorescence microscopy.

## Results and discussion

### Crystal structure of TiO_2_ nanotubes and surface characterization of PDA-immobilized nt-TiO_2_

**Figure 2:**
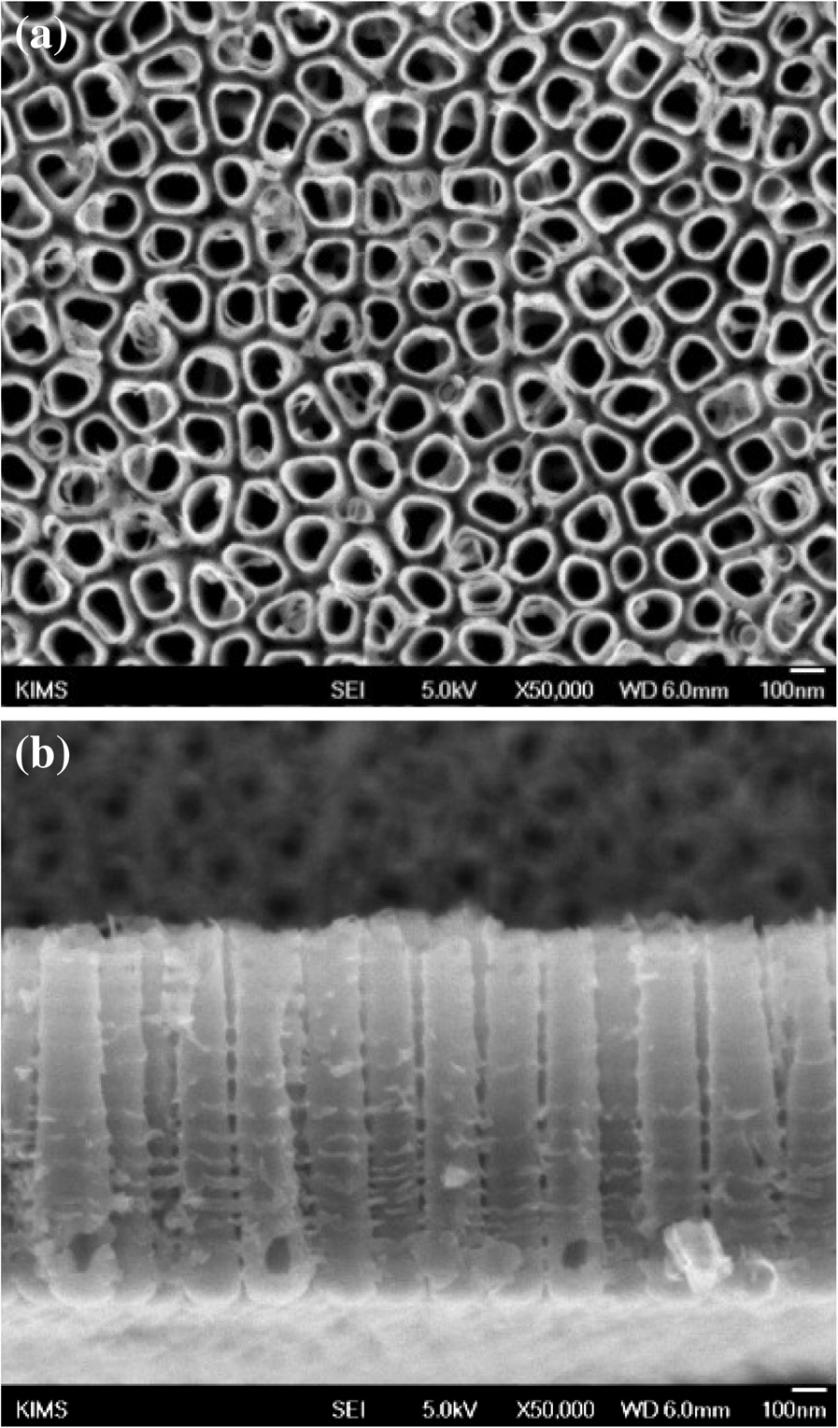
**Typical (a) surface and (b) cross-sectional FE-SEM images of TiO**_**2 **_**nanotubes.** The nanotubes were formed at an applied potential of 25 V for 2 h in 1 M H_3_PO_4_ + 0.3 M HF solution at 20°C.

**Figure 3:**
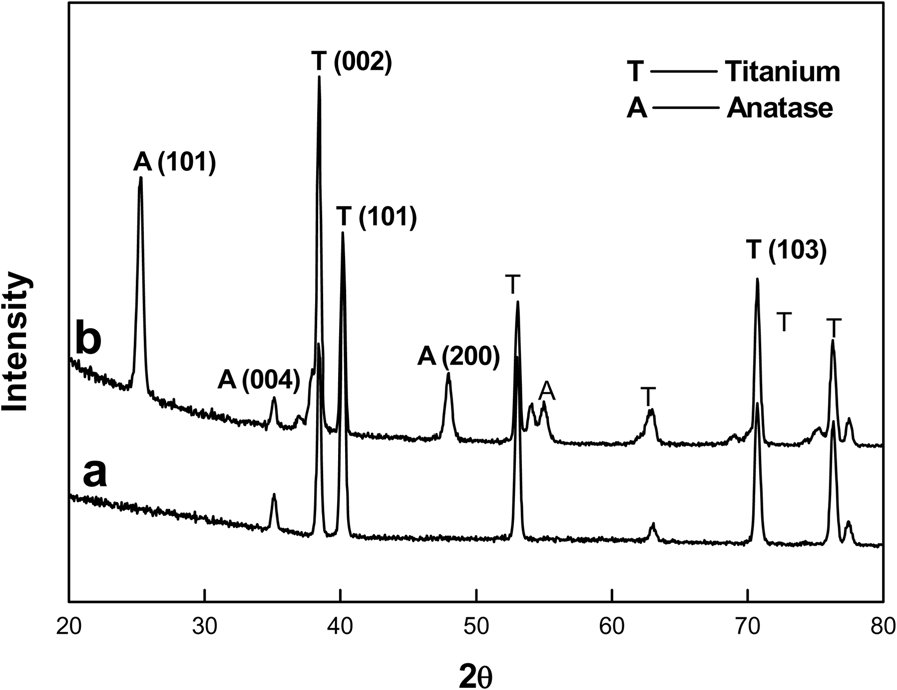
**XRD patterns of (a) Ti substrate and (b) heat-treated TiO**_**2 **_**nanotubes for 3 h at 350°C in air.** The nanotubes were formed at an applied potential of 25 V for 2 h in 1 M H_3_PO_4_ + 0.3 M HF solution at 20°C.

After anodization and annealing at 25 V and 350°C, respectively, the morphology of the highly ordered TiO_2_ nanotube array was examined by FE-SEM (Figure 2) to ascertain the nanotube dimensions. The mean outer diameters of the nanotubes were 100 nm. WAXD analysis (Figure 3) showed that the anodized nanotubes were amorphous, which transformed to anatase after heat treatment at 350°C [29].

**Figure 4:**
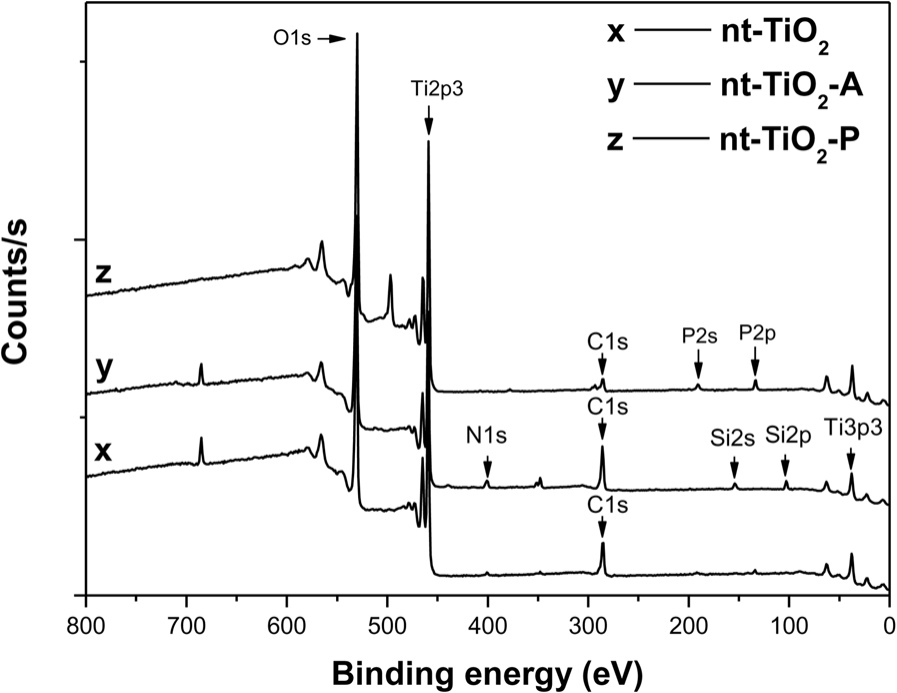
**XP spectra of nt-TiO**_**2 **_**(curve x), nt-TiO**_**2 **_**-A (curve y), and nt-TiO**_**2 **_**-P (curve z).**

**Figure 5:**
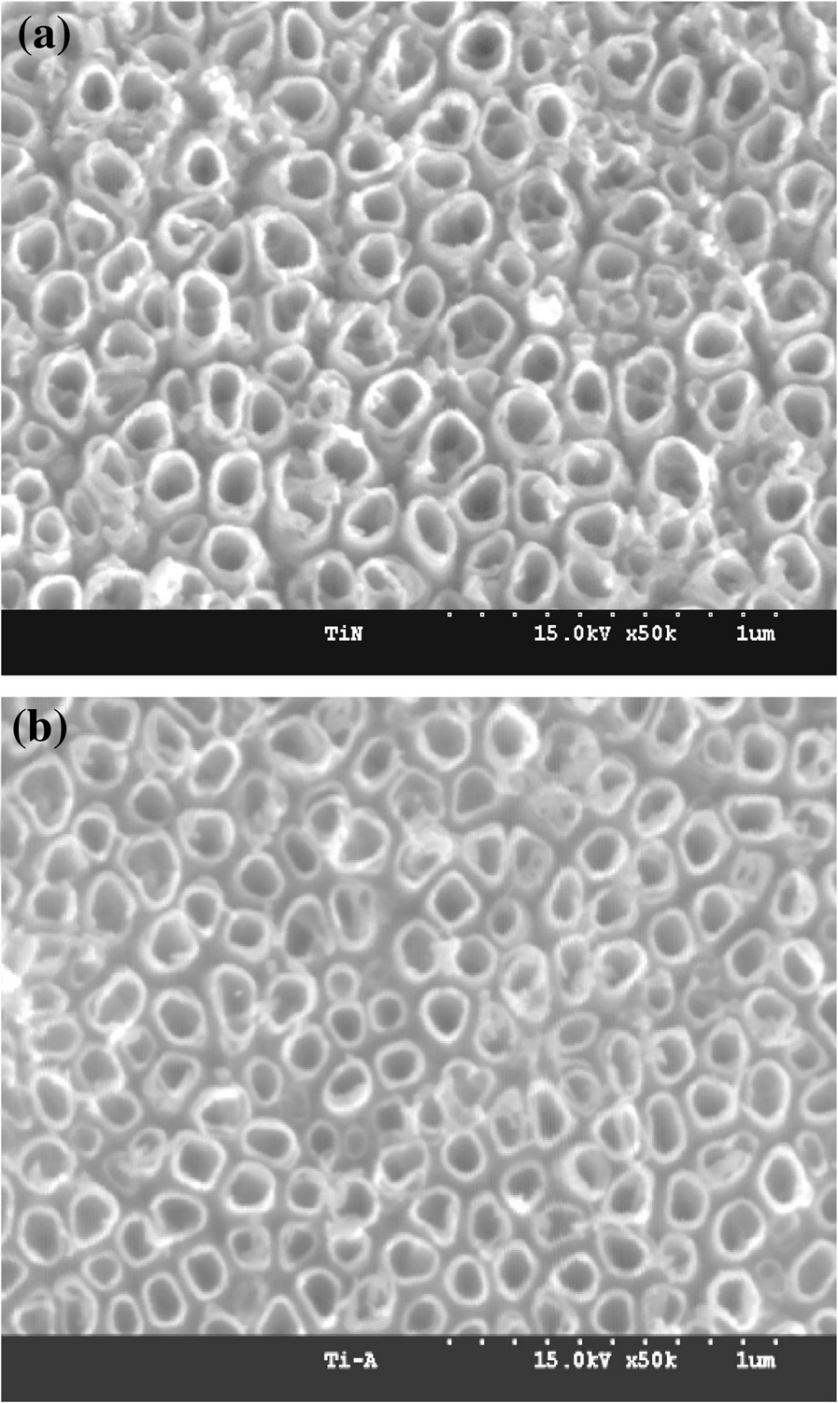
**FE-SEM images of (a) nt-TiO**_**2 **_**and (b) nt-TiO**_**2 **_**-P.**

ESCA was used to determine the immobilization of PDA on the nanotube surface (Figure 4). Table 1 lists the elements detected by quantitative analysis. The N 1*s* and P 2*p* photoelectron signal is the marker of choice for confirming PDA absorption. Three photoelectron signals were observed for nt-TiO_2_ (Figure 4, curve x) corresponding to C 1*s* (binding energy, 285 eV), Ti 2*p*^3^ (binding energy, 459 eV), and O 1*s* (binding energy, 529 eV). In contrast, five photoelectron signals were observed for nt-TiO_2_-A that correspond to C 1*s*, Ti 2*p*^3^, O 1*s*, N 1*s* (binding energy, 401 eV), and Si 2*s* (binding energy, 154 eV). On the other hand, one additional photoelectron signal was observed for nt-TiO_2_-P, which was assigned to P 2*p* (binding energy, 133.7 eV). The very weak N 1*s* photoelectron signal observed for nt-TiO_2_ might be due to the entrapment of atmospheric nitrogen and impurity. The binding energies of the N 1*s* and P 2*p* photoelectrons obtained from nt-TiO_2_-P were assigned to NH_2_^−^ (400.6 to 401.9 eV) and PO_4_^3−^ (133.7 eV), respectively [34]. The presence of two new elements, N and P, in nt-TiO_2_-P confirmed the absorption of PDA on the nanotube surface. The morphology of the TiO_2_ nanotubes was not significantly changed after immobilization of PDA (Figure 5).

**Table 1:** **Chemical composition of nt-TiO**_**2**_**and surface-modified nt-TiO**_**2**_

**Substrate**	**Atomic percent**					
	**O**	**C**	**Ti**	**N**	**Si**	**P**
nt-TiO_2_	56.4	22.2	20.5	0.9	-	-
nt-TiO_2_-A	49.9	27.5	16.3	3.2	3.1	-
nt-TiO_2_-P	58.3	16.1	21.6	1.3	0.8	1.9

### Interaction of bone cells with the surface-modified TiO_2_ nanotubes

#### Adhesion, proliferation, and differentiation of osteoblasts

**Figure 6:**
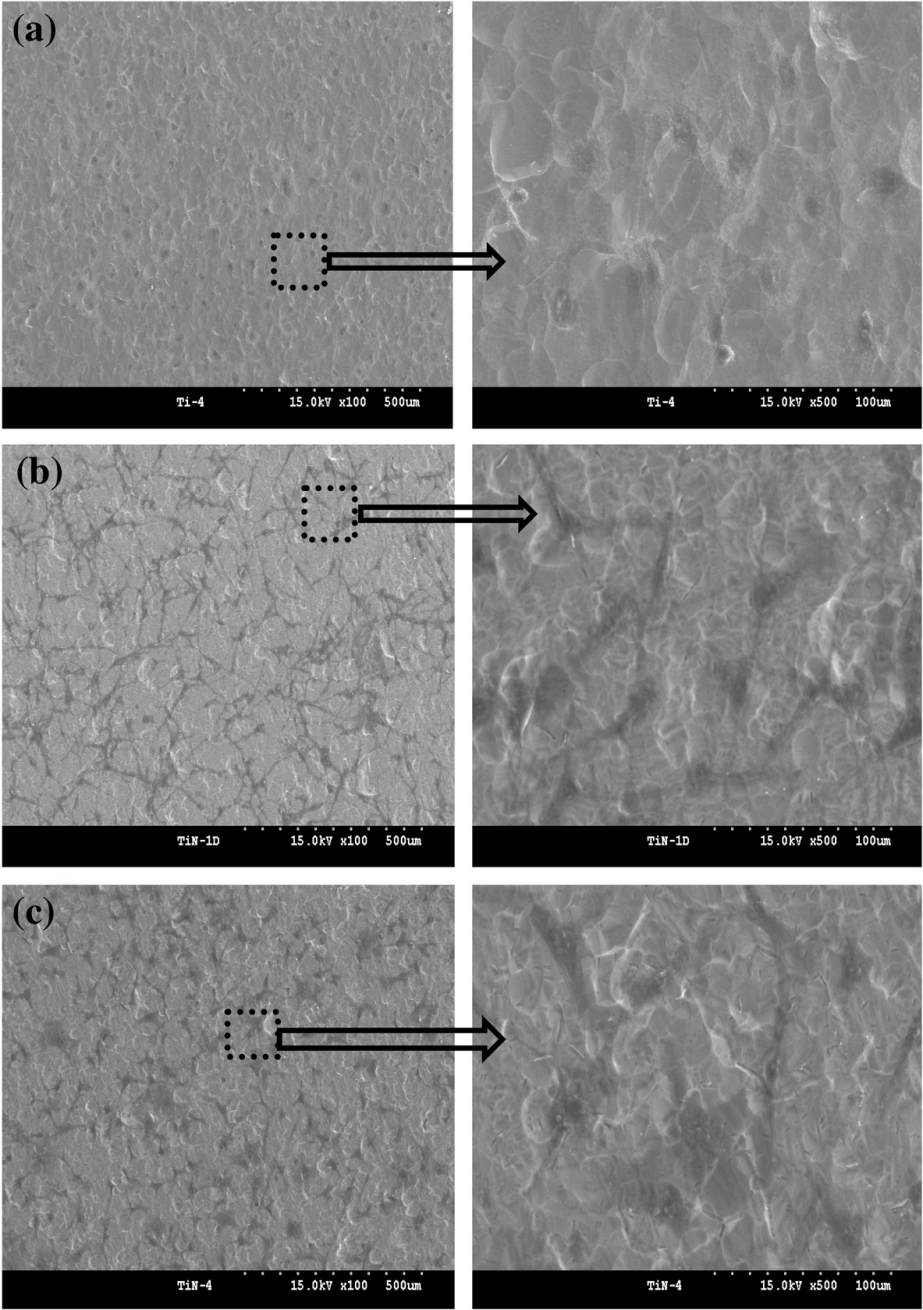
**FE-SEM images of adhering osteoblasts on (a) Ti, (b) nt-TiO**_**2 **_**, and (c) nt-TiO**_**2 **_**-P for 4 h.**

To examine the cell behavior on the unmodified and modified TiO_2_ surface, the osteoblasts were cultured on sand-blasted Ti, nt-TiO_2_, and nt-TiO_2_-P discs for 4 h and observed by FE-SEM (Figure 6). The osteoblast cells appeared as a dark phase in the FE-SEM image. After 4 h of culture, the osteoblast cells were mostly circular and barely spread on the Ti disc (Figure 6a). Osteoblast cell adhesion, spreading, and growth on the nt-TiO_2_ and nt-TiO_2_-P surfaces (Figure 6b,c) were enhanced compared to those on the control Ti disc, suggesting a good cell compatibility of nt-TiO_2_ and nt-TiO_2_-P.

**Figure 7:**
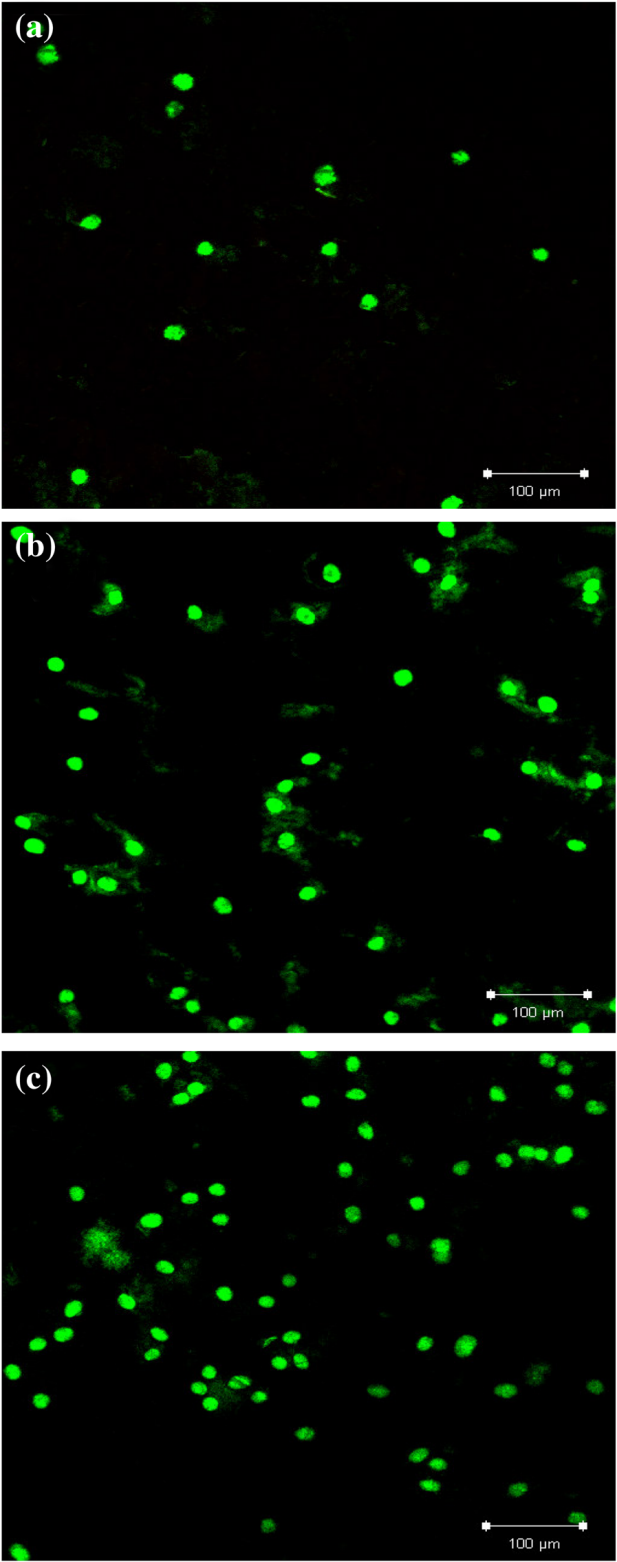
**Fluorescence microscopy images of osteoblast cells marked with calcein-AM (green) and propidium iodide (red).** The cells were cultured on **(a)** Ti, **(b)** nt-TiO_2_, and **(c)** nt-TiO_2_-P for 2 days.

Furthermore, the cytotoxic effect of PDA on osteoblast cells was analyzed by fluorescence microscopy using calcein-AM (green) and propidium iodide (red) as the markers which stain live and dead cells, respectively. Calcein-AM is highly lipophilic and cell membrane permeable. The calcein generated from the hydrolysis of calcein-AM by cytosolic esterase in a viable cell emits strong green fluorescence. Therefore, calcein-AM only stains viable cells. In contrast, propidium iodide, a nucleus-staining dye, can pass through only the disordered areas of the dead cell membrane and intercalates with the DNA double helix of the cell to emit a red fluorescence (excitation, 535 nm; emission, 617 nm). After 2 days of culture, green fluorescence areas were observed on all Ti, nt-TiO_2_, and nt-TiO_2_-P discs (Figure 7), suggesting the presence of live cells. A larger number of green fluorescence areas were identified on the nt-TiO_2_ and nt-TiO_2_-P discs (Figure 7b,c) than on the Ti discs (Figure 7a), indicating that the proliferation of osteoblasts was accelerated on nt-TiO_2_ and nt-TiO_2_-P than on the Ti disc. The absence of red fluorescence in nt-TiO_2_-P (Figure 7c) suggests that the immobilized PDA does not have any cytotoxic effect on osteoblast cells.

**Figure 8:**
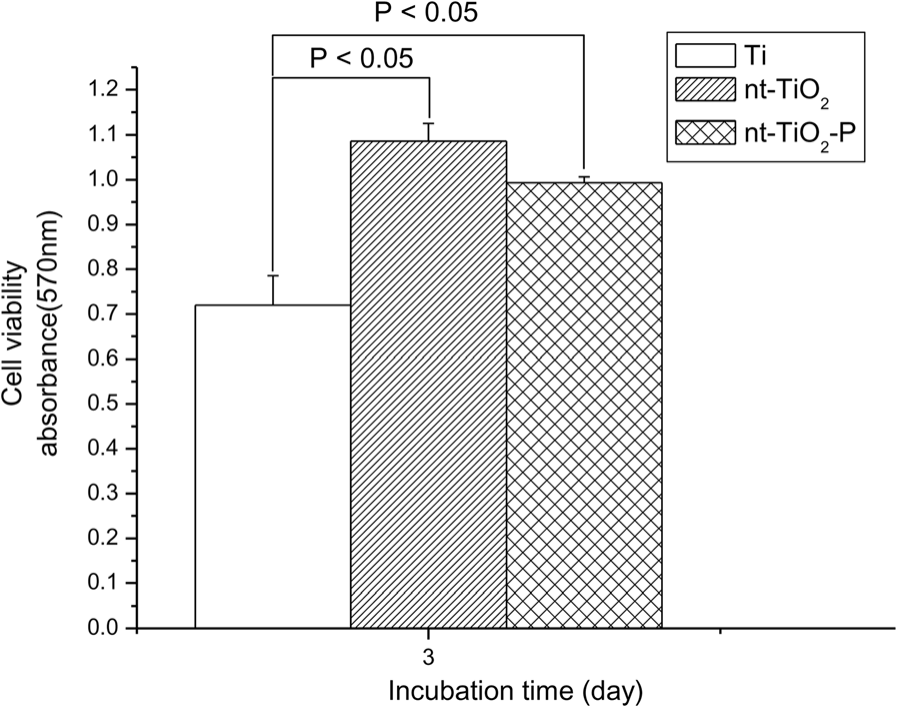
**MTT assay with absorbance as a measure of cell proliferation from osteoblast cells.** The cells were cultured on Ti, nt-TiO_2_, and nt-TiO_2_-P for different culture times.

The viability of osteoblast cells on Ti, nt-TiO_2_, and nt-TiO_2_-P discs at 3 days was analyzed by MTT assay. Cell proliferation on the nt-TiO_2_ and nt-TiO_2_-P discs was significantly (*P* < 0.05) higher than that on the Ti disc (Figure 8) after 3 days of culture. This suggests that nt-TiO_2_ and nt-TiO_2_-P provide a favorable surface for osteoblast adhesion and proliferation. The high osteoblast adhesion and proliferation on nt-TiO_2_ and nt-TiO_2_-P were attributed to the discrete nanostructure of the disc surface with gaps between the adjacent nanotubes (Figure 2). This typical subdivision structure minimized the interfacial stresses between the nanotube surface and osteoblasts and can allow the passage of body fluid that supplies the nutrients for cell growth. Moreover, vertically aligned TiO_2_ nanotubes have much larger surface areas than a flat Ti surface and contribute to the interlocked cell configuration [27,29].

**Figure 9:**
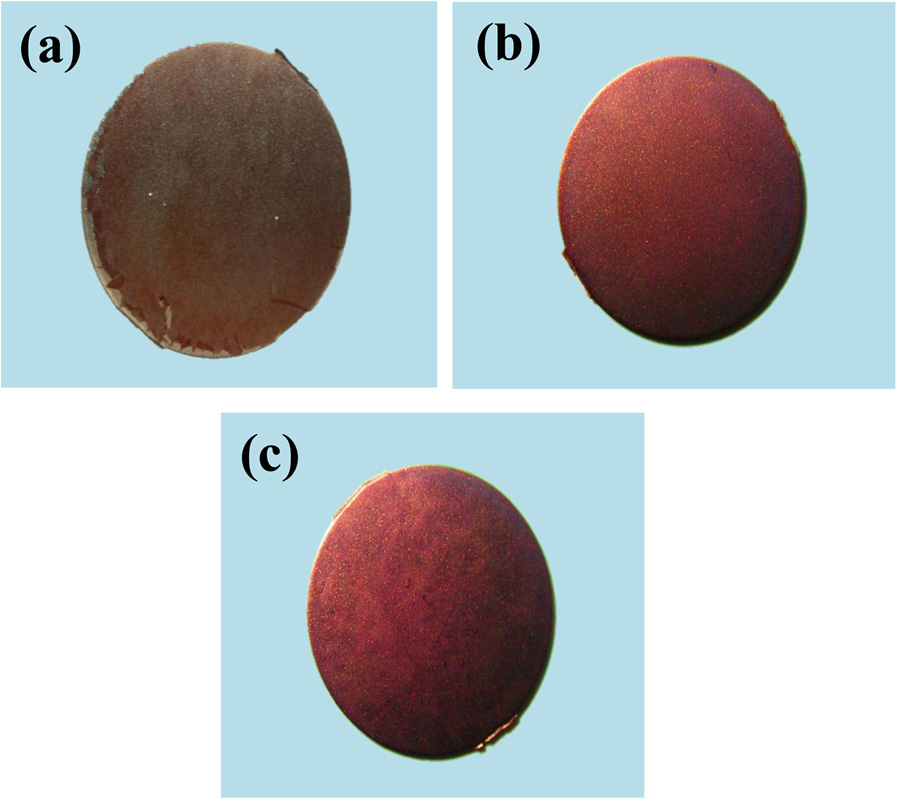
**Alizarin Red S staining of MC3T3-E1 osteoblasts.** The cells were cultured on **(a)** Ti, **(b)** nt-TiO_2_, and **(c)** nt-TiO_2_-P for 15 days: the calcium-containing area was stained in red.

Differentiation of osteoblast cells is one of the key processes for bone regeneration [35]. The *in vitro* differentiation of MC3T3-E1 into osteoblast phenotype was qualitatively observed by Alizarin Red S staining. Formation of bone nodule is one of the markers specific to bone cell differentiation. In the Alizarin Red S assay, calcification areas in the cells become stained in red. After staining with Alizarin Red S, intense dark red color was observed for the cells cultured on nt-TiO_2_ and nt-TiO_2_-P discs for 15 days (Figure 9b,c). However, the intensity of the red color is less for the cells cultured on the Ti disc (Figure 9a), suggesting that cells were differentiated more on the nt-TiO_2_ and nt-TiO_2_-P discs than on the Ti disc. These results mean that the nanotube structure is useful to accelerate the differentiation of osteoblasts.

#### Differentiation of macrophages into osteoclasts and viability on nanotube surface

**Figure 10:**
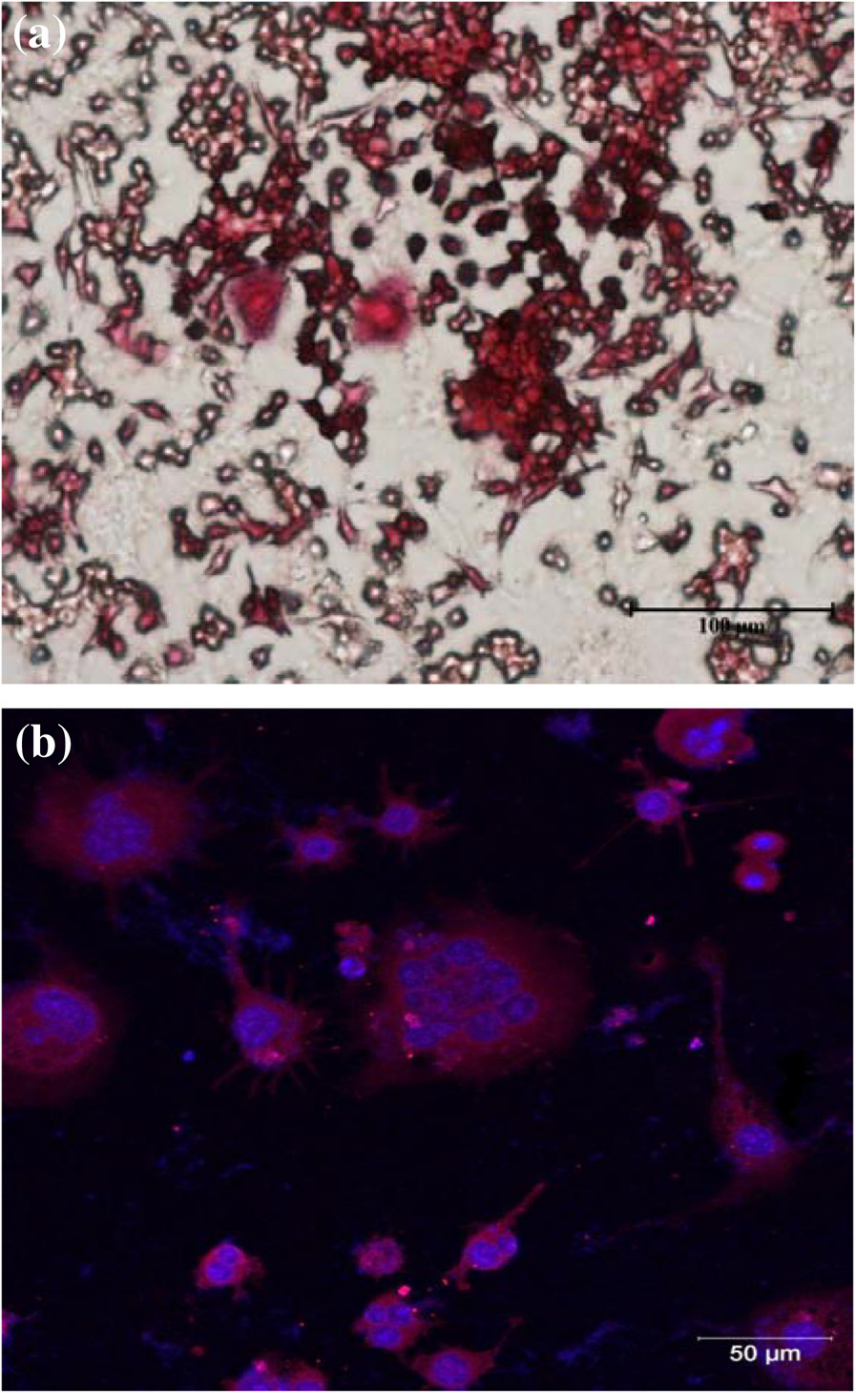
**Fluorescence microscopy images of (a) TRAP and (b) DAPI and phalloidin staining.** The macrophages differentiated into osteoclasts.

**Figure 11:**
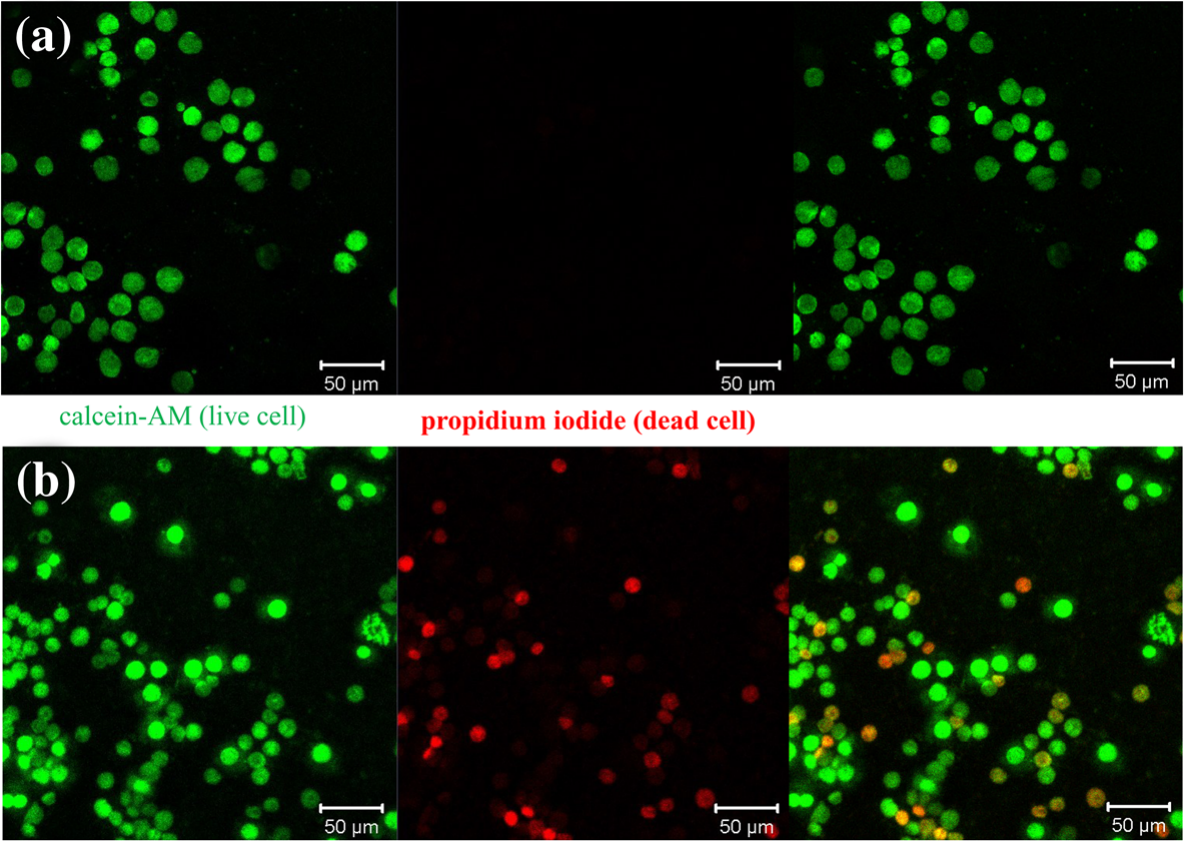
**Fluorescence microscopy images of calcein-AM (green) and propidium iodide (red).** The macrophages differentiated into osteoclasts on **(a)** nt-TiO_2_ and **(b)** nt-TiO_2_-P for 4 days.

To examine the viability of osteoclast cells on the PDA-immobilized nt-TiO_2_ surface, HSCs from mice were seeded on nt-TiO_2_ and nt-TiO_2_-P and induced to differentiate into multinucleated osteoclast-like cells using standard m-CSF and RANKL procedures. A series of markers were analyzed during the differentiation of the macrophage cells to osteoclasts. Tartrate-resistant acid phosphatase (TRAP) is a marker of osteoclasts and shows a red color when stained with tartrate and chromogenic substrate. TRAP-positive cells were observed as early as 4 days of differentiation (Figure 10). After 4 days of differentiation, more than 50% of the macrophages differentiated into osteoclasts. Furthermore, the nucleus and actin were stained with DAPI (blue) and TRICK (red), respectively, to confirm the differentiation of the macrophages into osteoclasts. The presence of multinucleated giant cells (osteoclast cells) along with mononucleated macrophage cells suggests that macrophage cells were partially differentiated into osteoclasts (Figure 11).

On the nt-TiO_2_ surface, differentiated osteoclasts stained with calcein-AM and propidium iodide showed a green color indicating the good viability of the cells. In contrast, along with green fluorescence, red fluorescence was also observed on the nt-TiO_2_-P surface, which suggests that some osteoclast cells died in contact with PDA (immobilized PDA did not show any cytotoxic effect on macrophage cells, Additional file 1: Figure S1). Osteoclasts normally destroy themselves by apoptosis, a form of cell suicide. PDA encourages osteoclasts to undergo apoptosis by binding and blocking the enzyme farnesyl diphosphate synthase in the mevalonate pathway [36]. Thus, the viability of osteoclasts was suppressed on the nt-TiO_2_-P surface, leading to a decrease in bone resorption activity and an increase in osseointegration and bone maturation.

## Conclusion

TiO_2_ nanotubes were successfully fabricated on Ti surface, and pamidronic acids were immobilized on the TiO_2_ nanotube surface. The adhesion and proliferation of osteoblasts were accelerated on the TiO_2_ nanotubes and pamidronic acid-conjugated TiO_2_ nanotubes compared to the Ti disc only. Macrophages were partially differentiated into osteoclasts by the addition of RANKL and m-CSF. The viability of osteoclasts was suppressed on the pamidronic acid-conjugated TiO_2_ nanotubes. This study has demonstrated that immobilization of PDA might be a promising method for the surface modification of TiO_2_ nanotube for use as dental and orthopedic implants. An *in vivo* study will be necessary to evaluate the potential of pamidronic acid-conjugated TiO_2_ nanotube as a therapeutic bone implant.

## Competing interests

The authors declare that they have no competing interests.

## Authors’ contributions

T-HK and Z-CX carried out the pamidronic acid immobilization on the nt-TiO_2_ disc and the cell experiment. JSB analyzed the experimental data and drafted the manuscript. S-MM and YJ prepared the nt-TiO_2_ disc. I-KK conceived of the study and participated in its design and coordination. All authors read and approved the final manuscript.

## Supplementary Material

Additional file 1: Figure S1 Fluorescence microscopy images of macrophage cells (calcein-AM and propidium iodide stained) cultured on nt-TiO_2_-P. Click here for file
