# Supplementary figures and images for: Immobilization of pamidronic acids on the nanotube surface of titanium discs and their interaction with bone cells
Source: Nanoscale Res Lett. 2013 Mar 12;8(1):124. doi: 10.1186/1556-276X-8-124 (PMC3602675; doi:10.1186/1556-276X-8-124)

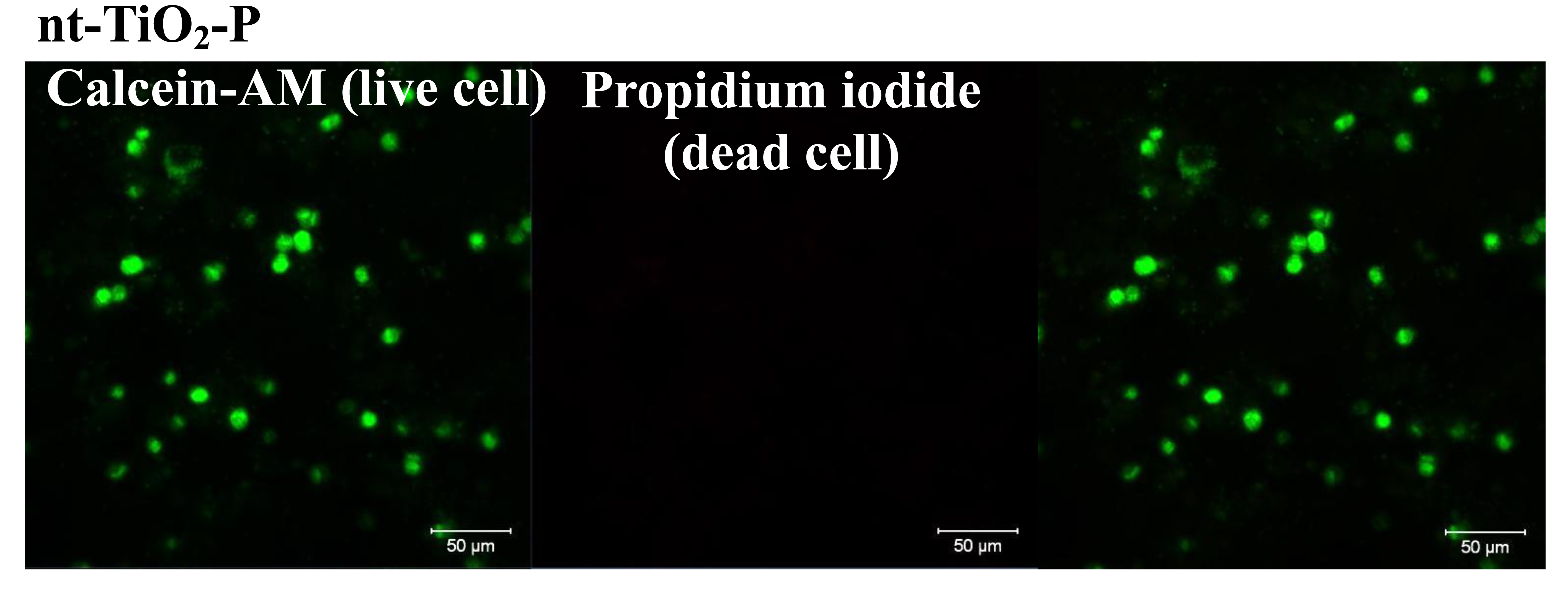

Supplement: Additional file 1: Figure S1 — Fluorescence microscopy images of macrophage cells (calcein-AM and propidium iodide stained) cultured on nt-TiO2-P. [file 1556-276X-8-124-S1.tiff]
